# Adjusted color probability codes for peripapillary retinal nerve fiber layer thickness in healthy Koreans

**DOI:** 10.1186/1471-2415-14-38

**Published:** 2014-03-29

**Authors:** Samin Hong, Sang-Myung Kim, Kyoungsoo Park, Jun Mo Lee, Chan Yun Kim, Gong Je Seong

**Affiliations:** 1 Institute of Vision Research, Department of Ophthalmology, Yonsei University College of Medicine, 50 Yonsei-ro, Seodaemun-gu, Seoul 120-752, Republic of Korea; 2 Siloam Eye Hospital, Seoul, Republic of Korea

**Keywords:** Glaucoma, Optical coherence tomography, Probability code, Retinal nerve fiber layer

## Abstract

**Background:**

Though a newly developed spectral domain optical coherence tomography (OCT) is at the center of interests for many ophthalmologic researchers and clinicians, its own characteristics are not fully evaluated yet. The main purpose of this study was to establish the adjusted color probability codes for peripapillary retinal nerve fiber layer (RNFL) thickness in healthy Koreans and to compare them with original color codes provided by spectral domain OCT.

**Methods:**

Two hundred ninety-five healthy Korean eyes were enrolled and their peripapillary RNFL thickness was measured by Cirrus OCT. For each decade of age, the normal thickness reference was determined on the basis of z-scores and the adjusted color probability codes were established. Then the agreements between adjusted and original color codes were calculated using weighted Kappa (K_w_) coefficient.

**Results:**

On the basis of K_w_ coefficient, the overall agreement between the adjusted and original probability color codes was not excellent (K_w_ range of 0.500 to 0.806). If the adjusted probability codes were assumed as a standard of comparison, the original color codes showed the false-negative in 11% of eyes and the false-positive in 0.3% of eyes for average RNFL thickness.

**Conclusions:**

Adjusted color probability codes judged by the Korean normative data showed a discrepancy with original codes. It implies that normal reference and adjusted probability codes for each ethnicity might be needed to determine whether a certain RNFL thickness is within normal range or not.

## Background

Due to the glaucomatous optic nerve damage and visual field loss often are asymptomatic in the early stages, the identification of individuals with glaucoma at the earliest possible time is important to prevent a calamitous loss in vision [1]. In the last decade, various new advanced technologies to assess the structural loss of retinal ganglion cell (RGC) axons have been introduced [2-11], one of which is optical coherence tomography (OCT) [12]. Using low coherence interferometry, it allows for noninvasive *in vivo* high-resolution cross-sectional tomographic retinal images and quantitative measurements of retinal nerve fiber layer (RNFL) thickness [13]. Since spectral domain OCT acquires real-time depth scans and recognizes them as a whole cube, it can provide three dimensional intraretinal imaging data from a single scan [14-16]. Even after scanning a fundus, clinicians can re-analyze the data in various ways. Although this seems to be an amazing new ophthalmic imaging device, its baseline characteristics must be evaluated before coming into use. In particular, we used the Cirrus OCT (Carl Zeiss Meditec, Inc., Dublin, CA) in this study [17,18].

Even in normal subjects, there are many factors including age, refractive error, ethnicity, axial length, and optic disc size may influence to the RNFL thickness [19-28]. However, when OCTs judge whether a certain RNFL thickness measurement is within normal range or not, they only take into consideration the subject’s age. Normal RNFL thickness ranges according to race are not available for the Cirrus OCT. In addition, although some report that the mean RNFL thickness of Asians may be thicker than that of Caucasians [24-29], their detailed information stratified by age and sectors is insufficient, especially for the Korean population.

In the present study, using the spectral domain Cirrus OCT, the Korean normative data of peripapillary RNFL thickness and the adjusted probability color codes were established. And the agreement between the adjusted and original probability codes was determined.

## Methods

### Subjects

After obtaining the approval of the Institutional Review Board, 166 healthy Korean subjects (age, 20 to 65 years old) who visited the Health Promotion Center of Gangnam Severance Hospital, Yonsei University College of Medicine, Seoul, Republic of Korea between September and October, 2008 were enrolled in this study. All study protocol adhered to the tenets of the Declaration of Helsinki. They underwent a comprehensive medical examination (including an ophthalmologic exam) and their clinical records were retrospectively reviewed. The subjects were excluded if they had any history of ocular trauma or intraocular surgical or laser treatment. All participants with diabetes or any other systemic disease or medication affecting the visual field or RNFL were also excluded.

### Ophthalmologic examination

The comprehensive ophthalmologic exam included corrected visual acuity (CVA), intraocular pressure (IOP), spherical and cylindrical refractive errors (Auto Ref-Keratometer RK-3, Canon, Inc., Tokyo, Japan), nonmydriatic fundus and optic disc photographs (Fundus Camera VX-10, Kowa Company, Ltd., Tokyo, Japan). According to the examination guidelines of our Health Promotion Center, CVA was defined as visual acuity in the habitual glass state when it was equal to or better than 20/25; when it was worse than 20/25, CVA was defined as the best-corrected visual acuity after the manifested refraction. IOP was checked with noncontact pneumotonometry (Tonometer TX-10, Canon, Inc.) first, and was re-checked with Goldmann applanation tonometry when repeated measurements were higher than 21 mmHg.

Peripapillary RNFL thickness was measured by an Optic Disc Cube 200 × 200 scan of the spectral domain Cirrus OCT (Model 4000, Software version 3.0.0.64, Carl Zeiss Meditec Inc.) without pupil dilation. The same instrument was used by the same operator. Scans with blinks or with low signal strength (less than six) were excluded from the analysis.

Only healthy eyes with a CVA of 20/30 or better, an IOP of below 21 mmHg, a spherical refractive error within +/− 4.00 diopters and a cylinder refractive error within +/− 3.00 diopters, and normal appearance of the optic nerve head, RNFL and fundus were included the study. Both eyes of each subject were included if they satisfied the entry criteria.

### Statistical analysis

**Figure 1:**
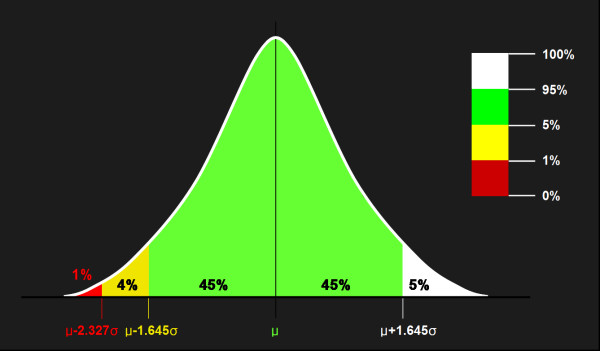
**Adjusted probability codes obtained on the basis of each z-score for a one-tailed normal probability of 5% (z = 1.645) and 1% (z = 2.327).** μ = mean; σ = standard deviation.

To establish the Korean normative dataset of peripapillary RNFL thickness for each decade of age, the mean and standard deviation of RNFL thickness were calculated for each scanned sector. After confirming the Korean normal distribution of RNFL thickness, the adjusted probability codes for Koreans were defined on the basis of z-score for a one-tailed normal probability of 5% (z = 1.645) and 1% (z = 2.327) (Figure 1). These adjusted probability codes for Koreans were compared with the original color probability codes, which were provided by built in analysis of Cirrus OCT, using the weighted Kappa (K_w_) coefficient using quadratic weights.

Calculation of the K_w_ coefficients was performed using the MedCalc program for Windows, version 9.6.4.0 (MedCalc Software, Mariakerke, Belgium), and all other statistical analyses were performed using SPSS for Windows, version 12.0.1 (SPSS Inc., Chicago, IL).

## Results

Two hundred ninety-five eyes of 166 healthy Korean subjects were analyzed, and their characteristics are shown in Table 1. The average age was 43.86 ± 10.33 years old (range 20 to 65). Thirty-seven eyes were excluded due to blinks during image scanning (7 eyes), low signal strength of the OCT image (3 eyes), poor CVA (14 eyes), and abnormal appearance of the optic nerve head or fundus (13 eyes). Although the CVA was worse in the 3^rd^ decade group than the 4^th^ decade group, all other characteristics except age and CVA were similar according to group.

**Table 1:** Subjects’ characteristics stratified by decade of age

	**Decade**					***p*-value**
	**3rd (20 – 29 yrs)**	**4th (30 – 39 yrs)**	**5th (40 – 49 yrs)**	**6th (50 – 59 yrs)**	**7th (60 – 65 yrs)**	
Number of eyes	27	83	90	75	20	-
Age (yrs)	26.70 ± 2.93	35.31 ± 2.33	44.13 ± 2.74	54.03 ± 3.24	63.15 ± 1.66	<0.001*
Gender (M : F)	15 : 12	49 : 34	38 : 52	44 : 31	14 : 6	0.071
Laterality (RE : LE)	14 : 13	40 : 43	46 : 44	38 : 37	9 : 11	0.982
CVA (LogMAR)	0.050 ± 0.061	0.021 ± 0.056	0.041 ± 0.085	0.067 ± 0.086	0.074 ± 0.081	0.002*
IOP (mmHg)	14.78 ± 2.90	14.41 ± 3.05	14.53 ± 2.83	14.59 ± 2.70	14.50 ± 2.24	0.983

CVA = corrected visual acuity; F = female; IOP = intraocular pressure; LE = left eye; M = male; RE = right eye.

Values are mean ± standard deviation.

*p < 0.05.

**Figure 2:**
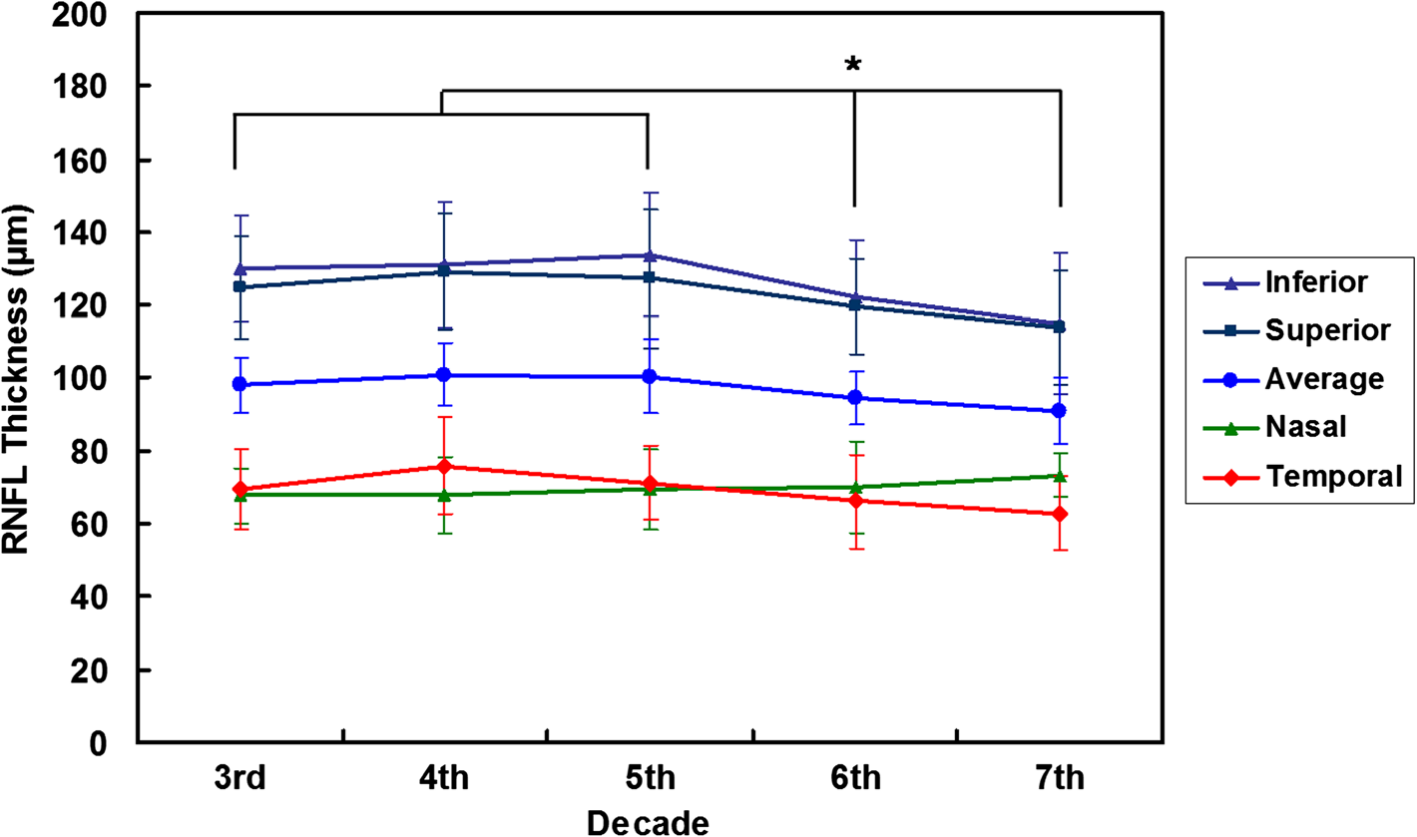
**Peripapillary retinal nerve fiber layer thickness according to age in healthy Koreans.** Asterisks indicate statistically significant differences (p < 0.050).

The mean RNFL thicknesses for each scanned sector and decade of age are shown in Table 2 and Figure 2. Overall, average RNFL thickness progressively decreased with age (Pearson’s correlation coefficient ρ, −0.251; p < 0.001). Superior, inferior, and temporal RNFL thickness also decreased with age (ρ range of −0.237 to −0.230; all p < 0.001), while nasal RNFL thickness did not (ρ, 0.140; p = 0.016).

**Table 2:** Peripapillary retinal nerve fiber layer thickness in healthy Koreans

**Sector**	**Decade**	Thickness* (μm)	**95% Confidence interval (μm)**	**Range (μm)**	***ρ***^**§**^	***p*-value**
Average	3^rd^ (20 – 29 yrs)	97.96 ± 7.51	94.99 – 100.93	85 – 112	−0.251 (SE, 0.053)	<0.001
	4^th^ (30 – 39 yrs)	101.01 ± 8.53	99.15 – 102.87	74 – 119		
	5^th^ (40 – 49 yrs)	100.47 ± 10.10	98.35 – 102.58	80 – 129		
	6^th^ (50 – 59 yrs)	94.59 ± 7.40	92.88 – 96.29	79 – 112		
	7^th^ (60 – 65 yrs)	91.10 ± 9.19	86.80 – 95.40	76 – 104		
	Total	98.26 ± 9.27	97.199 – 99.32	74 – 129		
Superior	3^rd^ (20 – 29 yrs)	124.82 ± 14.02	119.27 – 130.36	103 – 156	−0.237 (SE, 0.051)	<0.001
	4^th^ (30 – 39 yrs)	129.19 ± 15.84	125.73 – 132.65	94 – 173		
	5^th^ (40 – 49 yrs)	127.21 ± 19.04	123.22 – 131.20	77 – 185		
	6^th^ (50 – 59 yrs)	119.64 ± 13.06	116.64 – 122.65	81 – 144		
	7^th^ (60 – 65 yrs)	113.70 ± 15.70	106.36 – 121.05	84 – 143		
	Total	124.71 ± 16.68	122.80 – 126.62	77 – 185		
Inferior	3^rd^ (20 – 29 yrs)	130.00 ± 14.55	124.24 – 135.76	104 – 153	−0.230 (SE, 0.055)	<0.001
	4^th^ (30 – 39 yrs)	130.90 ± 17.22	127.14 – 134.66	94 – 176		
	5^th^ (40 – 49 yrs)	133.90 ± 17.11	130.32 – 137.48	99 – 175		
	6^th^ (50 – 59 yrs)	122.24 ± 15.88	118.59 – 125.89	59 – 153		
	7^th^ (60 – 65 yrs)	114.90 ± 19.10	105.96 – 123.84	76 – 136		
	Total	128.45 ± 17.62	126.43 – 130.47	59 – 176		
Temporal	3^rd^ (20 – 29 yrs)	69.41 ± 10.83	65.12 – 73.69	57 – 114	−0.233 (SE, 0.061)	<0.001
	4^th^ (30 – 39 yrs)	75.88 ± 13.18	73.00 – 78.76	49 – 112		
	5^th^ (40 – 49 yrs)	71.24 ± 10.29	69.09 – 73.40	51 – 101		
	6^th^ (50 – 59 yrs)	66.17 ± 12.83	63.22 – 69.12	43 – 136		
	7^th^ (60 – 65 yrs)	62.85 ± 10.19	58.08 – 67.62	51 – 88		
	Total	70.52 ± 12.50	69.09 – 71.95	43 – 136		
Nasal	3^rd^ (20 – 29 yrs)	67.74 ± 7.56	64.75 – 70.73	51 – 82	0.140 (SE, 0.052)	0.016
	4^th^ (30 – 39 yrs)	68.10 ± 10.41	65.82 – 70.37	51 – 93		
	5^th^ (40 – 49 yrs)	69.29 ± 10.88	67.01 – 71.57	46 – 99		
	6^th^ (50 – 59 yrs)	69.89 ± 12.42	67.04 – 72.75	27 – 99		
	7^th^ (60 – 65 yrs)	73.30 ± 5.82	70.58 – 76.03	60 – 81		
	Total	69.24 ± 10.68	68.01 – 70.46	27 – 99		

*Values are mean ± standard deviation.

^§^Pearson’s correlation coefficient between age and retinal nerve fiber layer thickness.

SE = standard error.

**Figure 3:**
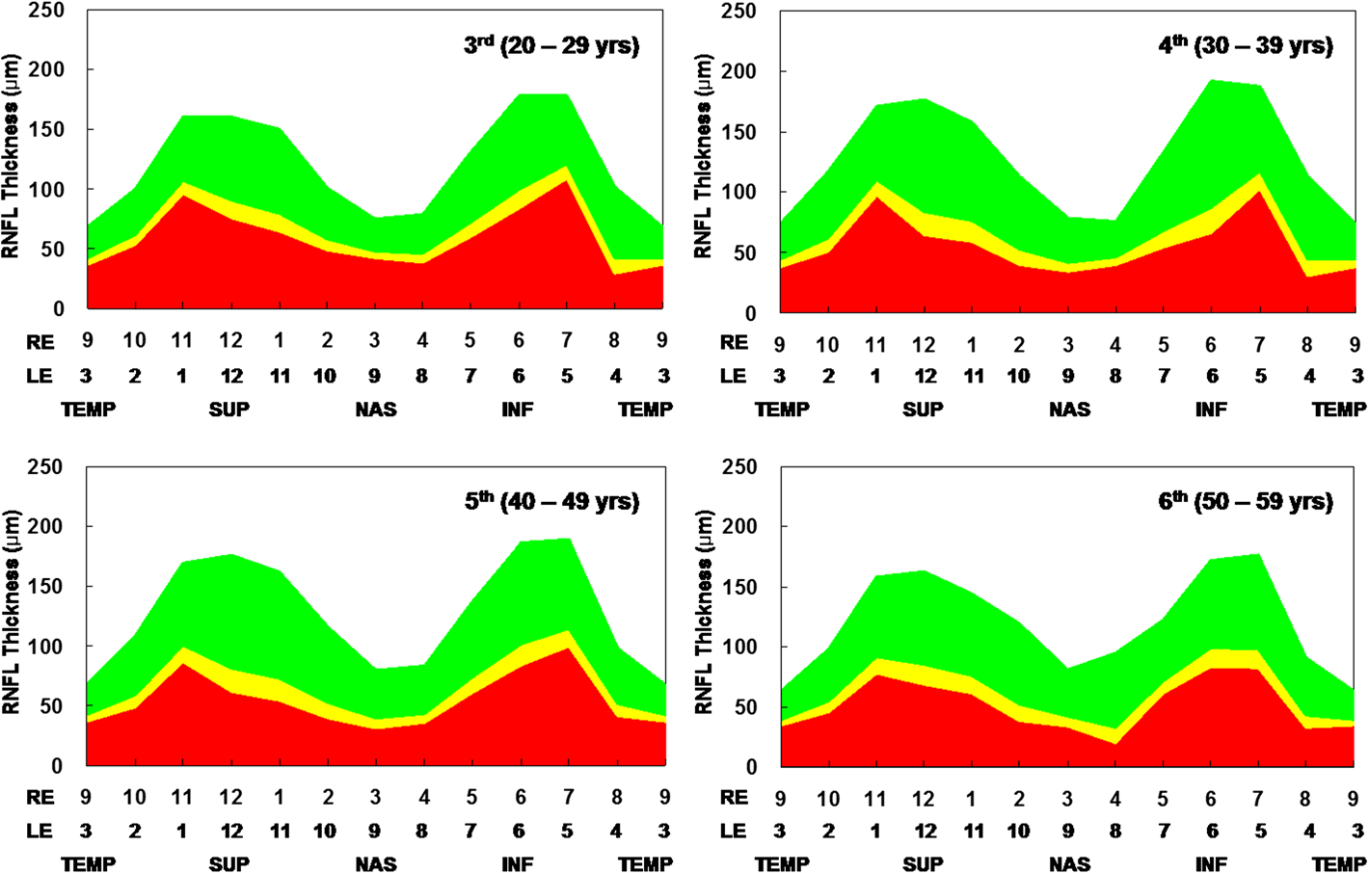
Normal distributions of retinal nerve fiber layer thickness in each decade of age for the Korean population.

Reference ranges of adjusted probability color codes for Koreans established on the basis of z-scores were generated. Values for the average and quadrants are shown in Table 3; values for the clock-hour scanned sectors are provided in Table 4 and Figure 3.

**Table 3:** Reference ranges of retinal nerve fiber layer thickness for adjusted probability codes for Korean population

	**Decade**	**Red code (0 – 1% probability)**	**Yellow code (1 – 5% probability)**	**Green code (5 – 95% probability)**	**White code (95 – 100% probability)**
Average	3^rd^ (20 – 29 yrs)	< 80.50	80.50 – 85.62	85.62 – 110.31	110.31 <
	4^th^ (30 – 39 yrs)	< 81.17	81.17 – 86.98	86.98 – 115.04	115.04 <
	5^th^ (40 – 49 yrs)	< 76.96	76.96 – 83.85	83.85 – 117.08	117.08 <
	6^th^ (50 – 59 yrs)	< 77.36	77.36 – 82.41	82.41 – 106.77	106.77 <
	7^th^ (60 – 65 yrs)	< 69.71	69.71 – 75.98	75.98 – 106.22	106.22 <
Superior	3^rd^ (20 – 29 yrs)	< 92.19	92.19 – 101.75	101.75 – 147.88	147.88 <
	4^th^ (30 – 39 yrs)	< 92.34	92.34 – 103.14	103.14 – 155.25	155.25 <
	5^th^ (40 – 49 yrs)	< 82.92	829.2 – 95.90	95.90 – 158.82	158.52 <
	6^th^ (50 – 59 yrs)	< 89.25	89.25 – 98.15	98.15 – 141.13	141.13 <
	7^th^ (60 – 65 yrs)	< 77.18	77.18 – 87.88	87.88 – 139.52	139.52 <
Inferior	3^rd^ (20 – 29 yrs)	< 96.14	96.14 – 106.07	106.07 – 153.93	153.93 <
	4^th^ (30 – 39 yrs)	< 90.84	90.84 – 102.58	102.58 – 159.23	159.23 <
	5^th^ (40 – 49 yrs)	< 94.09	94.09 – 105.76	105.76 – 162.04	162.04 <
	6^th^ (50 – 59 yrs)	< 85.30	85.30 – 96.13	96.13 – 148.35	148.35 <
	7^th^ (60 – 65 yrs)	< 70.47	70.47 – 83.49	83.49 – 146.31	146.31 <
Temporal	3^rd^ (20 – 29 yrs)	< 44.21	44.21 – 51.59	51.59 – 87.22	87.22 <
	4^th^ (30 – 39 yrs)	< 45.21	45.21 – 54.20	54.20 – 97.56	97.56 <
	5^th^ (40 – 49 yrs)	< 47.29	47.29 – 54.31	54.31 – 88.18	88.18 <
	6^th^ (50 – 59 yrs)	< 36.33	36.33 – 45.08	45.08 – 87.27	87.27 <
	7^th^ (60 – 65 yrs)	< 39.14	39.14 – 46.09	46.09 – 79.61	79.61 <
Nasal	3^rd^ (20 – 29 yrs)	< 50.14	50.14 – 55.30	55.30 – 80.18	80.18 <
	4^th^ (30 – 39 yrs)	< 43.87	43.87 – 50.97	50.97 – 85.23	85.23 <
	5^th^ (40 – 49 yrs)	< 43.97	43.97 – 51.39	51.39 – 87.19	87.19 <
	6^th^ (50 – 59 yrs)	< 40.99	40.99 – 49.46	49.46 – 90.32	90.32 <
	7^th^ (60 – 65 yrs)	< 59.75	59.75 – 63.72	63.72 – 82.88	82.88 <

Unit, μm.

**Table 4:** Reference ranges of clock-hour retinal nerve fiber layer thickness for adjusted probability codes for Korean population

	**Decade**	**Red code (0 – 1% probability)**	**Yellow code (1 – 5% probability)**	**Green code (5 – 95% probability)**	**White code (95 – 100% probability)**
CH9, RE/CH3, LE	3^rd^ (20 – 29 yrs)	< 37.28	37.28 - 42.59	42.59 - 68.22	68.22 <
	4^th^ (30 – 39 yrs)	< 38.05	38.05 - 44.28	44.28 - 74.32	74.32 <
	5^th^ (40 – 49 yrs)	< 37.48	37.48 - 42.64	42.64 - 67.50	67.50 <
	6^th^ (50 – 59 yrs)	< 34.74	34.74 - 39.66	39.66 - 63.40	63.40 <
	7^th^ (60 – 65 yrs)	< 33.95	33.95 - 38.43	38.43 - 60.07	60.07 <
CH10, RE/CH2, LE	3^rd^ (20 – 29 yrs)	< 53.93	53.93 - 61.77	61.77 - 99.57	99.57 <
	4^th^ (30 – 39 yrs)	< 51.23	51.23 - 62.45	62.45 - 116.54	116.54 <
	5^th^ (40 – 49 yrs)	< 49.29	49.29 - 59.38	59.38 - 108.06	108.06 <
	6^th^ (50 – 59 yrs)	< 45.90	45.90 - 54.89	54.89 - 98.23	98.23 <
	7^th^ (60 – 65 yrs)	< 43.00	43.00 - 51.55	51.55 - 92.75	92.75 <
CH11, RE/CH1, LE	3^rd^ (20 – 29 yrs)	< 96.58	96.58 - 107.57	107.57 - 160.58	160.58 <
	4^th^ (30 – 39 yrs)	< 98.00	98.00 - 110.47	110.47 - 170.64	170.64 <
	5^th^ (40 – 49 yrs)	< 86.88	86.88 - 101.10	101.10 - 169.68	169.68 <
	6^th^ (50 – 59 yrs)	< 77.71	77.71 - 91.49	91.49 - 157.95	157.95 <
	7^th^ (60 – 65 yrs)	< 71.41	71.41 - 84.45	84.45 - 147.35	147.35 <
CH12, RE/CH12, LE	3^rd^ (20 – 29 yrs)	< 76.11	76.11 - 90.55	90.55 - 160.19	160.19 <
	4^th^ (30 – 39 yrs)	< 64.95	64.95 - 84.00	84.00 - 175.93	175.93 <
	5^th^ (40 – 49 yrs)	< 61.98	61.98 - 81.52	81.52 - 175.75	175.75 <
	6^th^ (50 – 59 yrs)	< 69.38	69.38 - 85.45	85.45 - 163.00	163.00 <
	7^th^ (60 – 65 yrs)	< 64.36	64.36 - 80.23	80.23 - 156.77	156.77 <
CH1, RE/CH11, LE	3^rd^ (20 – 29 yrs)	< 65.26	65.26 - 79.82	79.82 - 150.04	150.04 <
	4^th^ (30 – 39 yrs)	< 59.68	59.68 - 76.48	76.48 - 157.54	157.54 <
	5^th^ (40 – 49 yrs)	< 54.43	54.43 - 72.97	72.97 - 162.43	162.43 <
	6^th^ (50 – 59 yrs)	< 61.79	61.79 - 75.91	75.91 - 144.01	144.01 <
	7^th^ (60 – 65 yrs)	< 63.64	63.64 - 76.32	76.32 - 137.48	137.48 <
CH2, RE/CH10, LE	3^rd^ (20 – 29 yrs)	< 49.01	49.01 - 57.98	57.98 - 101.28	101.28 <
	4^th^ (30 – 39 yrs)	< 40.44	40.44 - 52.86	52.86 - 112.80	112.80 <
	5^th^ (40 – 49 yrs)	< 40.15	40.15 - 53.20	53.20 - 116.18	116.18 <
	6^th^ (50 – 59 yrs)	< 38.53	38.53 - 52.48	52.48 - 119.76	119.76 <
	7^th^ (60 – 65 yrs)	< 55.83	55.83 - 64.67	64.67 - 107.33	107.33 <
CH3, RE/CH9, LE	3^rd^ (20 – 29 yrs)	< 42.58	42.58 - 48.10	48.10 - 74.72	74.72 <
	4^th^ (30 – 39 yrs)	< 34.71	34.71 - 42.27	42.27 - 78.76	78.76 <
	5^th^ (40 – 49 yrs)	< 31.44	31.44 - 39.65	39.65 - 79.28	79.28 <
	6^th^ (50 – 59 yrs)	< 33.92	33.92 - 41.96	41.96 - 80.74	80.74 <
	7^th^ (60 – 65 yrs)	< 47.66	47.66 - 53.60	53.60 - 82.30	82.30 <
CH4, RE/CH8, LE	3^rd^ (20 – 29 yrs)	< 39.13	39.13 - 45.94	45.94 - 78.80	78.80 <
	4^th^ (30 – 39 yrs)	< 40.38	40.38 - 46.37	46.37 - 75.29	75.29 <
	5^th^ (40 – 49 yrs)	< 35.84	35.84 - 43.96	43.96 - 83.11	83.11 <
	6^th^ (50 – 59 yrs)	< 20.18	20.18 - 32.96	32.96 - 94.61	94.61 <
	7^th^ (60 – 65 yrs)	< 53.10	53.10 - 56.81	56.81 - 74.69	74.69 <
CH5, RE/CH7, LE	3^rd^ (20 – 29 yrs)	< 60.08	60.08 - 72.25	72.25 - 130.94	130.94 <
	4^th^ (30 – 39 yrs)	< 54.73	54.73 - 68.27	68.27 - 133.56	133.56 <
	5^th^ (40 – 49 yrs)	< 60.65	60.65 - 73.75	73.75 - 136.94	136.94 <
	6^th^ (50 – 59 yrs)	< 61.50	61.50 - 71.95	71.95 - 122.34	122.34 <
	7^th^ (60 – 65 yrs)	< 59.86	59.86 - 68.94	68.94 - 112.76	112.76 <
CH6, RE/CH6, LE	3^rd^ (20 – 29 yrs)	< 83.80	83.80 - 99.93	99.93 - 177.77	177.77 <
	4^th^ (30 – 39 yrs)	< 66.30	66.30 - 87.77	87.77 - 191.32	191.32 <
	5^th^ (40 – 49 yrs)	< 84.45	84.45 - 101.92	101.92 - 186.19	186.19 <
	6^th^ (50 – 59 yrs)	< 83.95	83.95 - 99.02	99.02 - 171.70	171.70 <
	7^th^ (60 – 65 yrs)	< 58.66	58.66 - 77.75	77.75 - 169.85	169.85 <
CH7, RE/CH5, LE	3^rd^ (20 – 29 yrs)	< 109.26	109.26 - 121.04	121.04 - 177.85	177.85 <
	4^th^ (30 – 39 yrs)	< 103.28	103.28 - 117.61	117.61 - 186.75	186.75 <
	5^th^ (40 – 49 yrs)	< 99.86	99.86 - 115.18	115.18 - 189.13	189.13 <
	6^th^ (50 – 59 yrs)	< 82.63	82.63 - 98.73	98.73 - 176.42	176.42 <
	7^th^ (60 – 65 yrs)	< 60.09	60.09 - 80.62	80.62 - 179.68	179.68 <
CH8, RE/CH4, LE	3^rd^ (20 – 29 yrs)	< 30.05	30.05 - 42.32	42.32 - 101.53	101.53 <
	4^th^ (30 – 39 yrs)	< 30.64	60.64 - 44.80	44.80 - 113.12	113.12 <
	5^th^ (40 – 49 yrs)	< 41.93	41.93 - 51.64	51.64 - 98.51	98.51 <
	6^th^ (50 – 59 yrs)	< 33.42	33.42 - 43.25	43.25 - 90.69	90.69 <
	7^th^ (60 – 65 yrs)	< 31.26	31.26 - 41.73	41.73 - 92.27	92.27 <

CH = clock-hour.

Unit, μm.

The overall agreement between the adjusted and original probability color codes was not excellent [30,31]. The K_w_ coefficients for average, superior, inferior, temporal, nasal RNFL thickness were 0.500 (SE, 0.055), 0.729 (SE, 0.056), 0.642 (SE, 0.055), 0.803 (SE 0.057), and 0.806 (SE, 0.058), respectively.

When the adjusted probability codes were assumed as a standard of comparison, 11 eyes (3.7%) were false negative but no eye was false positive for average RNFL thickness (Table 5). The superior (Table 6), inferior (Table 7), and temporal (Table 8) quadrants showed similar tendencies of greater false negative rate than false positive rate. On the contrary, for the nasal quadrant, the false positive rate (7 cases, 2.4%) was greater than the false negative (1 case, 0.3%) (Table 9). Overall, if the adjusted probability color codes for Koreans were used as a standard of comparison, about 11% of eyes for average RNFL thickness measurement and about 7% of eyes for quadrant RNFL thickness measurement were judged differently from the original color codes. When the white and green codes were categorized as normal RNFL thickness, about 4% of eyes for average RNFL thickness measurement and about 2% of eyes for quadrant RNFL thickness measurement were judged as false negative.

**Table 5:** Agreement between adjusted and original probability codes for average retinal nerve fiber layer thickness

		**Original probability code**			**Total**
		**White/Green**	**Yellow**	**Red**	
Adjusted probability code	White/Green	284	-^§^	-^§^	284 (96.3%)
	Yellow	10^*^	-	-^§^	10 (3.4%)
	Red	-^*^	1^*^	-	1 (0.3%)
Total		294 (99.7%)	1 (0.3%)	0 (0.0%)	

^*^False negative. ^§^False positive.

Values are number of eyes (%).

**Table 6:** Agreement between adjusted and original probability codes for superior retinal nerve fiber layer thickness

		**Original probability code**			**Total**
		**White/Green**	**Yellow**	**Red**	
Adjusted probability code	White/Green	282	-^§^	-^§^	282 (95.6%)
	Yellow	3^*^	6	2^§^	11 (3.7%)
	Red	-^*^	1^*^	1	2 (0.7%)
Total		285 (96.6%)	7 (2.4%)	3 (1.0%)	

*False negative. ^§^False positive.

Values are number of eyes (%).

**Table 7:** Agreement between adjusted and original probability codes for inferior retinal nerve fiber layer thickness

		**Original probability code**			**Total**
		**White/Green**	**Yellow**	**Red**	
Adjusted probability code	White/Green	282	-^§^	-^§^	282 (95.6%)
	Yellow	4^*^	4	4^§^	12 (4.1%)
	Red	1^*^	-^*^	-	1 (0.3%)
Total		287 (97.3%)	4 (1.4%)	4 (1.4%)	

^*^False negative. ^§^False positive.

Values are number of eyes (%).

**Table 8:** Agreement between adjusted and original probability codes for temporal retinal nerve fiber layer thickness

		**Original probability code**			**Total**
		**White/Green**	**Yellow**	**Red**	
Adjusted probability code	White/Green	285	-^§^	-^§^	285 (96.6%)
	Yellow	7^*^	2	-^§^	9 (3.1%)
	Red	-^*^	1^*^	-	1 (0.3%)
Total		292 (99.0%)	3 (1.0%)	0 (0.0%)	

^*^False negative. ^§^False positive.

Values are number of eyes (%).

**Table 9:** Agreement between adjusted and original probability codes for nasal retinal nerve fiber layer thickness

		**Original probability code**			**Total**
		**White/Green**	**Yellow**	**Red**	
Adjusted probability code	White/Green	277	7^§^	-^§^	284 (96.3%)
	Yellow	1^*^	8	-^§^	9 (3.1%)
	Red	-^*^	-^*^	2	2 (0.7%)
Total		278 (94.2%)	15 (5.1%)	2 (0.7%)	
^*^FN	1 (0.3%)				
^§^FP	7 (2.4%)				

^*^False negative. ^§^False positive.

Values are number of eyes (%).

**Table 10:** Information about internal normartive data of spectral domain Cirrus OCT

**Ethnic group**	**Number of subjects (%)**	**Age category**	**Number of subjects (%)**
Caucasian	122 (43.0%)	18 – 29 yrs	60 (21.1%)
Hispanic	35 (12.3%)	30 – 39 yrs	53 (18.7%)
African American	51 (18.0%)	40 – 49 yrs	45 (15.8%)
Chinese	63 (22.2%)	50 – 59 yrs	53 (18.7%)
Korean	3 (1.1%)	60 – 69 yrs	42 (14.8%)
Indian	3 (1.1%)	> 70 yrs	31 (10.9%)
Japanese	1 (0.4%)		
Other	6 (2.1%)		
Total	284 (100.0%)	Total	284 (100.0%)

From Carl Zeiss Meditec, Inc., Dublin, CA.

## Discussion

In this study, Korean normative data of peripapillary RNFL thickness as measured by spectral domain Cirrus OCT were established, and the reference ranges of RNFL thickness for adjusted color probability codes for the Korean population were generated. Our healthy eyes were selected by normal appearance of the optic nerve head and retinal nerve fiber rather without perimetric test. At present, diagnosis of glaucoma generally requires evidence of typical glaucomatous optic nerve atrophy in the absence of other potential causes. Due to these include functional disturbance traditionally identified by visual field abnormalities and anatomical evidence of damage in optic nerve head and peripapillary retinal nerve fiber, it has been better to perform a standard automated perimetric test. However, many glaucoma researchers have suggested that defects in the appearance of the retinal nerve fiber may represent early, clinically detectable manifestations of glaucomatous damage [30-39]. They also widely agreed the careful assessment of optic nerve head and retinal nerve fiber might identify patients with glaucomatous neuropathy before development of reproducible field defects. Assessment of optic nerve head and retinal nerve fiber is valuable as an early indicator of glaucomatous optic neuropathy.

The average and three quadrants (all except the nasal quadrant) showed RNFL thickness that gradually thinned with age. Regarding the agreements between the adjusted and original color probability codes, the overall agreements were not excellent. For average RNFL thickness, the value of the K_w_ coefficient was just 0.500, which only indicates moderate agreement. Even for the superior and inferior RNFL thicknesses, the K_w_ coefficients were 0.729 and 0.642, respectively. Theoretically, since probability codes are based on a one-tailed normal probability of the entire population, probability codes adjusted for each ethnicity might be more useful than the original color codes judged by an internal database from mixed populations. In healthy Koreans, more than 10% of measurements were judged differently with the adjusted probability codes, and most were judged more seriously compared to the original codes.

Since the company (Carl Zeiss Meditec, Inc.) has not disclosed detailed information about the current internal database of Cirrus OCT, we can only know the proportion of each ethnic group and age category (Table 10). *Regarding ethnicity, the Cirrus OCT only includes the data of 122 Caucasian, 35 Hispanic, 51 African American, 63 Chinese, 3 Korean, 3 Indian, 1 Japanese, and 6 other subjects.* Even though the current built-in internal database in the Cirrus OCT includes more Asians than the time domain Stratus OCT (Carl Zeiss Meditec, Inc.) [23], normal RNFL thickness ranges according to race are not available for the Cirrus OCT. Additionally, for the Cirrus OCT, the normative RNFL thickness data stratified by age are not available for any ethnicity. Furthermore, except for Caucasians, only a small number of subjects are included for each ethnicity, and their age distribution is not uniform.

**Figure 4:**
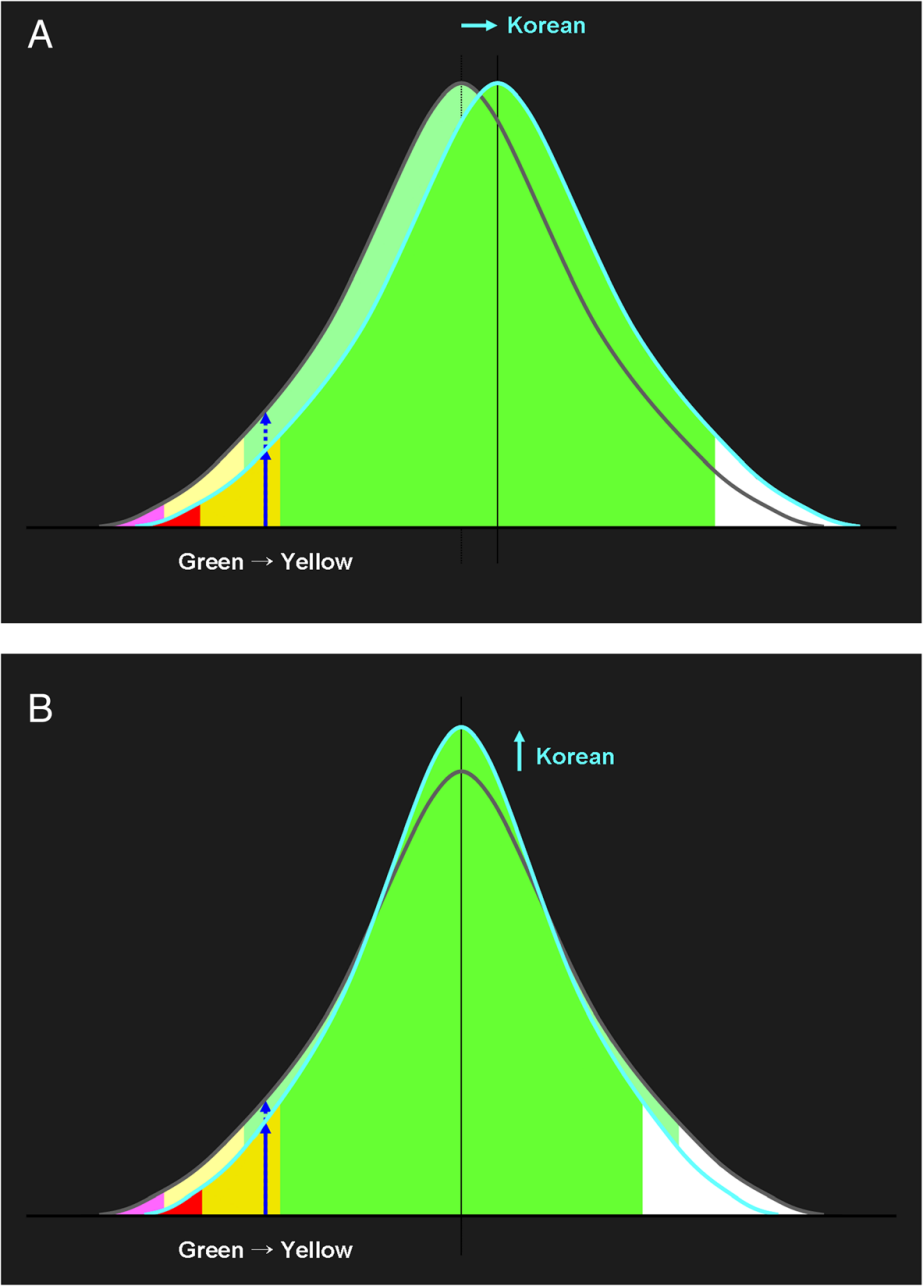
Theoretical causes of the discrepancy between adjusted and original probability codes: the means (A) or standard deviations (B) might be different.

Next, we consider reasons for the discrepancy between the adjusted and original color probability codes. Since the probability codes are based on z-scores, they are determined by the mean and standard deviation of RNFL thicknesses. Although exact information on the current internal database is not available, if the mean of Koreans is greater than the internal database, some probability codes should be adjusted (Figure 4A). However, even if the mean is not different, the standard deviation might be, in which case the probability codes should also be adjusted (Figure 4B). Generally, it might be better to use probability codes adjusted for each ethnicity rather than the original codes. The adjusted probability codes might be a great help not only to glaucoma specialists but also to general ophthalmologists when they face borderline cases. Furthermore, our data will be useful for comparing racial differences in normal RNFL thickness.

## Conclusion

The adjusted color probability codes for Koreans showed a discrepancy with the original color codes. Due to the normal reference ranges of peripapillary RNFL thicknesses and probability codes can differ according to race, the normative data might be needed for every ethnicity. When clinicians judge whether a certain RNFL thickness measurement is within normal limits or not, they need to take the race of the patient into consideration.

## Abbreviations

CVA: Corrected visual acuity; IOP: Intraocular pressure; Kw: Weighted Kappa; OCT: Optical coherence tomography; RGC: Retinal ganglion cell; RNFL: Retinal nerve fiber layer.

## Competing interest

None of authors has a financial competing interest.

## Authors’ contributions

SH and GJS designed the study; SH collected data and wrote the bulk of the manuscript; SMK, KP, and JML contributed to the data analysis; CYK and GJS revised the manuscript; GS has given final approval of the version to be published. All authors read and approved the final manuscript.

## Pre-publication history

The pre-publication history for this paper can be accessed here:

http://www.biomedcentral.com/1471-2415/14/38/prepub
